# Post-Traumatic Growth in Professionals Caring for People with Intellectual Disabilities during COVID-19: A Psychological Intervention

**DOI:** 10.3390/healthcare10010048

**Published:** 2021-12-28

**Authors:** María Inmaculada Fernández-Ávalos, María Nieves Pérez-Marfil, Manuel Fernández-Alcántara, Rosario Ferrer-Cascales, Francisco Cruz-Quintana, Oliver Hugh Turnbull

**Affiliations:** 1 Faculty of Health Sciences, Department of Health Psychology, University of Alicante, 03690 Alicante, Spain; inmaculada.fernandez@ua.es (M.I.F.-Á.); mfernandeza@ua.es (M.F.-A.); rosario.ferrer@ua.es (R.F.-C.); 2 Mind, Brain and Behavior Research Center (CIMCYC), University of Granada, 18071 Granada, Spain; fcruz@ugr.es; 3 End-of-Life Research Network (EOL), 18071 Granada, Spain; 4 School of Psychology Brigantia Building, Bangor University, Bangor LL57 2AS, UK; o.turnbull@bangor.ac.uk

**Keywords:** professionals, intellectual disability, COVID-19, mental health, psychological intervention, post-traumatic growth

## Abstract

Background: Health professionals present a greater vulnerability to the effects of COVID-19 on their mental health, especially those who work with vulnerable groups such as those who suffer from intellectual disability (ID). The objective of the present research was to develop and verify the effectiveness of a psychological intervention for professionals in the field of ID to improve their mental health during this health crisis. Methods: A total of 32 professionals participated. The variables measured were: post-traumatic growth, mental health, burnout, coping strategies, resilience, life satisfaction, optimism, and cognitive and affective empathy. Results: The results revealed statistically significant differences in the post-traumatic growth variable. In the rest of the variables (mental health, burnout, coping strategies, resilience, vital satisfaction, optimism, and empathy), no significant differences between groups were found. Conclusions: An increase in the levels of post-traumatic growth was observed in the intervention group after a brief online psychological intervention. However, given the small sample size, these results should be taken with caution. Institutions should foster and promote interventions aimed at reducing the high emotional impact produced by COVID-19 in professionals that care for people diagnosed with ID.

## 1. Introduction

COVID-19 (SARS-CoV-2) was first identified in December 2019 in Wuhan, where it spread rapidly to other territories and countries [1]. The pandemic rapidly became a major global threat, with devastating consequences for the global economy and public health [1,2], in which Spain was one of the main affected territories.

The pandemic was characterized by a high rate of infection with a range of effects on physical health, including age-variable rates of mortality [3,4]. In addition, it is important not to ignore the substantial impact that it has had on mental health [4,5]. Vindegaard and Benros [6] found that patients with COVID-19 had higher levels of post-traumatic stress and depressive symptoms, and a worsening of psychiatric symptoms in those with pre-existing disorders. In relation to the general world population, effects such as an increase in psychological discomfort, levels of anxiety, depression, stress and post-traumatic symptoms, a decrease in positive emotions, and sleep problems such as insomnia have been found [4,5,6].

Health professionals may present with a greater vulnerability to the effects of COVID-19 on their mental health, as they are a group exposed to infection in their work environment [7,8]. Previous studies have identified that health workers presented symptoms of depression, anxiety, stress, distress, poor sleep quality, insomnia, denial, anger, and fear [7,8,9,10]. These effects were prolonged in the medium and long term, producing post-traumatic stress disorders, anxiety, depression, and burnout, often at high levels of intensity [5,11].

Workers who provide care for people with intellectual disabilities (IDs) described having a great sense of responsibility for the vulnerable people they support. They also felt that they were a neglected group. Consistent with this, there is scant literature on their mental health [12,13]. When the topic was investigated, it was found that fear, frustration, disappointment, job stress, uncertainty about the role, and worsening mental well-being were the main emotions that these professionals felt during the COVID-19 pandemic [12,13]. However, they were proud of the perseverance and effort they put into their work, despite the difficulties [12,13]. Indeed, studies prior to COVID-19 suggested that this group already presented symptoms of stress, burnout, and depression [14].

While the impact on mental health of health workers by COVID-19 has been documented, there is little literature on the efficacy of interventions to address this issue. Investigations have typically used individual online therapies with a cognitive-behavioral orientation and some group therapies using psychoeducation techniques and mindfulness, with the aim of improving the regulation of emotions and reducing anxiety/stress and insomnia among healthcare staff [15,16,17,18,19,20,21,22,23]. In emergencies, telephone assistance has been used to provide immediate psychological support [24,25]. Mobile phone applications and online courses have also been proposed [22,24,26]. Although all these proposals appear to be promising, so far there are no clear results on their efficacy [10].

The present study aimed to develop and verify the effectiveness of a psychological intervention for professionals in the field of ID to improve their mental health during this health crisis.

The intervention of the present research was based on a group therapy approach that allows emotional expression and understanding between the different members of the team to manage the strong emotional crisis they were experiencing [27,28]. Likewise, a short cognitive-behavioral program based on mindfulness was taught, since in previous studies it has been observed that both types of therapy helped to reduce levels of anxiety, stress, and depression, regulate emotions, and improve psychological well-being [20,29,30]. Finally, the proposed intervention was carried out online to minimize the risk of contagion of COVID-19 [18].

## 2. Materials and Methods

### 2.1. Design

A quasi-experimental randomized study was performed on a group of professionals who cared for people with ID, divided into an intervention group (IG) and a control group (CG).

### 2.2. Participants

Participants were all professionals who worked with adults diagnosed with ID as defined by DSM-5 criteria [31]. All worked in an organization located in the province of Granada, Spain, that provides care for people with the diagnosis of ID. The organization offers daily drop-in and residential housing services, depending on the type of support and the situation of the person. The inclusion criteria were that participants: (a) had been working in the center for at least 1 year; (b) had daily contact with the users of the center; (c) worked a minimum weekly frequency of 3 days per week with the users; and (d) were working in the center at the time of the pandemic.

**Figure 1:**
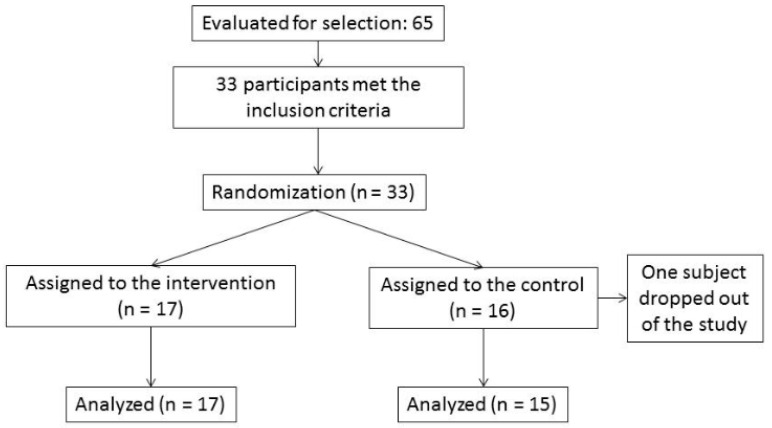
Flow of participants through the phases of a parallel randomized clinical trial of two groups.

At the time of the study, the center had 65 professional employees. Of these, 33 met the inclusion criteria (Figure 1). Participants were assigned to groups following simple randomization (1:1) using a computer-generated random number sequence: resulting in the IG = 17 and CG = 16. One participant (in the CG) dropped out of the study because of illness, so the final sample consisted of 32 professionals. The IG (N = 17, 3 men, 14 women) had a mean age of 41.1 years (SD = 11.07), and the CG (N = 15, 9 men, 6 women) had a mean age of 39.1 years (SD = 12.54).

The sociodemographic data for each group are shown in Table 1. In general, there were no statistically significant differences between the groups in the variables described above except for the gender, with the number of women in the IG being higher.

### 2.3. Instruments

Post-traumatic Growth Inventory Short form (PTGI-SF) [32]. This instrument evaluates the perception of personal benefits, or positive changes, in survivors of a traumatic event. We used a version adapted for the Spanish population [33] that consists of 10 items, with 6 Likert-type response options (“no change” to “very high degree of change”). Higher scores reflect greater perceived change. The Spanish version of the scale had adequate psychometric properties, with higher values in reliability (α = 0.83) [33]. In the present study the Cronbach’s α was 0.93.

Goldberg General Health Questionnaire (GHQ-28) [34]. This questionnaire assesses psychosocial health through 4 subscales: Somatic symptoms (Subscale A); Symptoms of anxiety/distress and insomnia (Subscale B); Symptoms of social dysfunction (Subscale C); and Symptoms of depression (Subscale D). We used the version validated for the Spanish population [35]. It consists of 28 items (7 items in each subscale) with 4 Likert-type response options. Higher scores reflect decreasing levels of mental health. This Spanish version of the scale had adequate values of internal consistency (α = 0.93) [35]. In the present study the Cronbach’s α was 0.84.

Maslach Burnout Inventory (MBI) [36]. This instrument measures the frequency and intensity of burnout through 3 subscales: emotional exhaustion (EE), depersonalization (DP), and personal accomplishment (PA). In this study, the scale validated for the Spanish population was used [37]. It consists of 22 items with a Likert-type response format that includes 7 response options (“never” to “every day”). High scores in the first two subscales and low scores in the third are indicative of burnout. The reliability values in the Spanish version of the scale were: α = 0.90 in EE, α = 0.79 in DP, and α = 0.71 in PA [37]. The reliability values calculated in the present study of the scale were: α = 0.82 in EE, α = 0.44 in DP and α = 0.68 in PA.

Connor–Davidson Resilience Scale (CD-RISC-10) [38]. This instrument is a measure of resilience. In the present study, we used the version adapted for the Spanish population [39], which consists of 10 items with a 5 response Likert-type format (“not at all” to “almost always”). Higher scores reflect greater resilience. In psychometric terms, this Spanish version had higher values in reliability (α = 0.85) [39]. The Cronbach’s α in the present study was 0.80.

Satisfaction With Life Scale (SLWS) [40]. This instrument assesses life satisfaction through a more subjective analysis. The version adapted for the Spanish population was used in this research [41] and consists of 5 items with 7 Likert-type response options (“totally disagree” to “totally agree”). Higher scores reflect greater perceived life satisfaction. Psychometric properties for this Spanish validation revealed adequate values of internal consistency (α = 0.84) [41]. The Cronbach’s α in the current study was 0.70.

Life Orientation Test Revised (LOT-R) [42]. This instrument assesses the level of dispositional optimism. The version adapted for the Spanish population was used in this study [43]. It consists of 10 items with 5 Likert-type response options (“totally disagree” to “totally agree”). Of these 10 items, 3 are written in the affirmative (1, 4 and 10), 3 are written in the negative (3, 7 and 9), and 4 do not contribute to the total score. Items that are written negatively are reverse coded. Higher scores reflect greater optimism. This Spanish version had adequate values of internal consistency (α = 0.75) [43]. In the present study the Cronbach’s α was 0.44.

Cognitive and Affective Empathy Test (TECA) [44]. This instrument evaluates ‘overall’ empathy, as well as 2 cognitive sub-components (Perspective adoption and Emotional Understanding) and 2 affective sub-components (Empathic Stress, and Empathic Joy). In the present study, only the overall Empathy score (in its original Spanish version) was used [44]. This consists of 33 items, on a 5 response Likert-type scale (“totally disagree” to “totally agree”). Higher scores reflect higher levels of empathy. In psychometric terms, this Spanish scale had adequate values of internal consistency (α = 0.86) for the overall TECA scores [44]. In the present study the Cronbach’s α was 0.60. 

Coping Strategies Inventory (CSI) was also included [45,46]. This instrument evaluates different aspects of coping through 8 subscales: Problem Solving (PS), Self-criticism (SC), Express emotion (EE), Wishful Thinking (DT), Social Support (SS), Cognitive Restructuring (CR), Problem Avoidance (PA) and Social Withdrawal (SW) [45,46]. The version validated into Spanish version was used in this research [46], which consisting consists of 40 items with 4 Likert-type response options (“not at all” to “totally”). The Spanish validation presented a α adaptation has a Cronbach’s alpha between 0.63 and 0.89 in its different subscales [46]. The reliability values calculated in our study presented a α between 0.60 and 0.94 in its different subscales

### 2.4. Psychological Intervention

An intervention program entitled *“Professionals in emotional crisis: COVID-19”* was developed based on a cognitive-behavioral paradigm [20,29,30]. The psychological techniques most used for the identification and management of emotions were focused on elements of mindfulness-based techniques: ventilation and emotional management, meditation, self-compassion, and self-care. Likewise, to promote the subjective well-being of professionals by identifying and controlling their negative and irrational thoughts, psychological techniques such as cognitive restructuring, psychoeducation, and self-instruction were used. These techniques followed well-established guidelines for action and suggestions for recommended activities [29,47,48]. The mapping of these across sessions is outlined in Table 2. The main objectives of this program were based on managing and accepting the new situation experienced, as well as promoting optimal coping strategies to overcome the obstacles presented by the COVID-19 situation.

The program was delivered in four weekly sessions (each approximately 1 h). These sessions (delivered by the same qualified psychologist) were carried out in two groups: one of 8 and one of 9 professionals. Both the intervention and the evaluations were carried out online. Each session had a similar structure: (a) a brief reminder of the previous session; (b) development of the objective of the session; (c) activities related to the session content; (d) resolution of doubts; and (e) summary and final conclusions.

The sessions were carried out with simple oral presentations, accompanied by audio-visual material such as photos and videos.

In the case of the CG, no topic was worked on. After finishing the psychological intervention, the CG was contacted to offer them the possibility to participate in the program. However, owing to time limitations none of the participants in the CG decided to take part in the psychological intervention.

### 2.5. Procedure

The study was carried out between November and December 2021. Before starting the study, the researchers met personally with the directors and managers of the association, and an informative meeting was held in order to explain the purpose of the research.

A meeting was then scheduled with the professionals from the center who met the inclusion criteria to inform them of the characteristics of the research, the objectives of the study, and to request their collaboration. The professionals who agreed to participate received a document with information about the study and signed an informed consent. No professional refused to participate, and participants did not receive remuneration. Omission of names or other identifying data helped ensure confidentiality. The study was approved by the Human Research Ethics Committee of the University of Granada (Ref: 445/CEIH/2017).

The pre- and post-intervention evaluations in the IG and CG were carried out using the instruments described above. All evaluations (duration approximately 30 min) were carried out online, individually.

### 2.6. Data Analysis

The quantitative data were analyzed using IBM SPSS for Windows v.22.0. Between-group differences were analyzed using *t* tests (for independent samples) and χ^2^. Linear models for repeated measures (Wilks’ lambda) were used to evaluate the effect of the program. Levels for the between-group factors were IG and CG. The two intra-subject levels were pre- and post-intervention. In all cases, the assumptions of homogeneity of variances (Levine’s test) were taken into account. Effect size was calculated with Cohen’s *d*. Statistical significance was set at *p* < 0.05. In addition, considering the large number of comparisons, the Benjamini–Hochberg Adjusted *p*-value [49] was calculated for the Time x Group interactions [50].

## 3. Results

Table 3 presents the means, standard deviations, effect sizes (Cohen’s *d*), and the results obtained from the differences between groups, the evaluation time, and the interactions between the different variables. The dependent variables presented correspond to burnout, cognitive empathy, resilience, post-traumatic growth, life satisfaction, optimism, coping strategies, and mental health.

As can be seen in Table 3, most of the findings were non-significant. The only variable that showed a statistically significant change was that of Post-traumatic growth, with a significant difference in the interaction of Time x Group (F(1,30) = 5.998; *p* = 0.021). These results reflected a substantial increase (20.1–27.2, roughly 0.5 s.d.) before and after the intervention in the IG. The CG showed a small decline across this period (29.2–27.9). No significant differences were found for the individual factors of Time or Group.

On the subscales of mental health, no significant differences were found in any of the factors (Time, Group, Time x Group). It is of note that there was a decrease (8.5–6.9) on the Somatic subscale, though this did not reach significance. For the three dimensions of the burnout variable (Emotional Exhaustion, Depersonalization, and Personal Accomplishment), there were no significant differences in any of the factors (Time, Group, Time x Group). We noted that the IG did have lower mean scores on Depersonalization after the intervention, compared to the CG, though this effect did not reach significance. For resilience, life satisfaction, optimism, and cognitive empathy, there were no significant differences for any of the factors (Time, Group, Time x Group). Here, the mean scores showed no substantial differences (in either group) pre- or post-evaluation.

In relation to the dimensions of the coping strategies no significant differences were found in any of the factors (Time, Group, Time x Group). On the dimensions of Self-criticism (*F*(1) = 6.307; *p* = 0.019) and Express Emotion (*F*(1) = 5.278; *p* = 0.030), there were significant differences in the Time factor, with a slight increase in the mean scores in both groups in the pre- and post-evaluations. On the rest of the dimensions the scores remained similar.

However, in general, the effect sizes (Cohen’s *d*) were low. In addition, when applying the Benjamini–Hochberg Adjusted *p*-value to the Time x Group interactions, the statistical significance of the results was remarkably reduced.

## 4. Discussion

The objective of this research was to evaluate the effectiveness of a psychological intervention aimed at professionals working in the field of ID to improve their mental health during the COVID-19 crisis.

First, comparing the overall results of the IG and CG, we found that significant differences were only seen in the post-traumatic growth variable: that is, the participants who benefited from the intervention had increased scores on this variable but no others. Post-traumatic growth refers to the positive change that a person experiences as the result of a struggle after experiencing a traumatic event [32,33]. Given that the participants in this research experienced a traumatic situation, it was expected that they experienced changes in their self-confidence, self-esteem, interpersonal relationships, spirituality and/or religiosity, and vision of the world in a similar way to that found in previous research [51]. In fact, most of the participants during the intervention felt that COVID-19 was one of the most difficult situations they had experienced, but from which they learned to value aspects of their life and work that previously went unnoticed. Therefore, since the roles of positive and negative emotions and beliefs are related to post-traumatic growth [51,52], work on emotional regulation and the promotion of positive psychology can help the individual to build a more resilient personality and thus better cope with future situations like the pandemic [29].

Other measures that did not reveal significant differences after the intervention were mental health-related dimensions (somatic, anxiety, social dysfunction, and depression). Here the means decreased in the IG, especially in the somatic dimension, but the changes did not reach significance. While not statistically significant, these results were broadly in line with the impact that COVID-19 has made on the mental health of health workers, as shown in previous research [7,8,9,10]. Naturally, the professionals in our study (and in other fields that work with vulnerable populations) risk developing mental health problems related to the substantial pressure to which they are subjected because they must protect and care for both the service users and themselves [12,53,54,55]. This raises the question of why these mental health problems did not produce a larger number of sequelae [5,11]. This resilience (discussed below) may relate to strategies such as practicing sports, leisure, occupational tasks other than the work environment, and receiving formal and informal social support [15,28], all of which might help professionals to disconnect from the loop of negativity in which they find themselves [27,28].

In relation to burnout, the professionals in this study typically expressed feeling overwhelmed by the demands of their work and the care required by the people with ID that they assisted. In addition, they admitted to the fear of contracting the virus and spreading it to their loved ones, which were similar to concerns found in health professionals from other fields [54,55]. However, all our participants maintained similar scores in the pre- and post-evaluation, which may relate to their levels of work stress during COVID-19. Burnout is a state of emotional exhaustion that does not arise in a short period of time, nor might it be expected to be overcome rapidly [11,36,37]. Therefore, it is reasonable to think that, just as its gestation is slow and gradual over time, its treatment also needs more time and practice to alleviate it, especially in situations of high emotional impact such as COVID-19 [16,54,55]. Strategies such as reducing workload, improving schedules, and organizing using mindfulness techniques can help prevent and/or reduce burnout in these workers [20,29,30].

Finally, and in relation to the above, despite the professionals having experienced negative emotions because of the difficulties experienced during the pandemic, their levels of resilience, life satisfaction, and optimism remained stable in both evaluations. The role of these protective factors seems essential in cushioning the stress endured and in promoting the mental well-being of these workers [30,51,52,56]. Specifically, some studies have reported that these positive personality traits may protect against powerful emotions such as fear, depression, stress, and anxiety [52,56]. Consequently, gratitude, self-compassion, self-confidence, and creativity are elements of positive psychology that professionals can practice in their daily lives to strengthen their mental health [30,52,56]. Likewise, the professionals appeared to have gained high levels of cognitive and affective empathy since the study began, reflecting a great interest in understanding and helping the population they serve [44]. In principle, this can be beneficial for a good worker–user relationship [53], although health care professionals must also be cautious that their professional involvement does not lead to emotional fatigue [11,36,37].

The results of our research have important clinical and practical implications, since again it was observed that vulnerable populations, such as people with ID, suffer many consequences from critical situations such as the COVID-19 pandemic [12,57,58,59]. In fact, the study by Cuschieri and Grech [57] observed that those with non-communicable diseases had a 6.55% higher risk of COVID-19 infection and often required hospital admission, mainly because of their vulnerability. Therefore, the COVID-19 pandemic offers us the opportunity to promote health and social policies in vulnerable groups through interdisciplinary and biopsychosocial approaches, and indeed international collaboration, and the opportunity to understand where to act and what to modify. As a result, these vulnerable people will not be more affected in future critical situations because of their challenging conditions, diagnoses, or chronic diseases [57,58,59].

The study has strengths and limitations. Regarding strengths, it analyzed the effectiveness of a brief psychological intervention program aimed at professionals who work in the field of ID, to improve their mental health during COVID-19. This was evaluated through a quantitative analysis that examined several variables related to the problems caused by the pandemic and the professionals’ approach to facing it. This program offered several potential benefits: (a) fostering optimal coping strategies for future problematic situations; (b) promoting higher levels of adherence to the program, as were applicable to professionals from various fields; (c) needing only limited material or professional resources for implementation and start-up; and (d) promoting social support among the workers who participated. In addition, data were collected by comparing an IG and a CG to increase the validity of the study.

Regarding the limitations, firstly, the sample was small and was recruited from a single center, so a generalization of the results requires caution. Secondly, when statistical corrections were applied, the *p*-value of the interactions decreased, so further research in a large sample is needed to verify the present results. Thirdly, the CG did not receive a placebo intervention and, owing to time limitations, members of this group did not want to participate in the psychological intervention after the end of the study. Fourthly, as we have already seen, the pandemic involved many changes to daily life, and a brief intervention may not lead to major changes in a few sessions. Finally, it was not possible to obtain a longer-term follow-up of participants.

These limitations reflect the challenges faced by those who design interventions for this population. Clearly, there is a need for support of health workers in these challenging settings. However, they also lack the time and energy to take part in the sort of long-lasting programs that might be of the greatest assistance to them. In addition, those allocated to any control or placebo conditions lack the time and energy to dedicate to a less time-demanding intervention. Solutions to this dilemma may involve better screening of participants into different groups, based on availability and enthusiasm, and perhaps a self-paced approach that allows participants to manage dosage levels themselves.

## 5. Conclusions

In conclusion, an increase in the levels of post-traumatic growth was observed in the intervention group after a brief online psychological intervention. In the other variables (mental health, burnout, coping strategies, resilience, vital satisfaction, optimism, and empathy), no significant differences were found, although trends moved in the appropriate direction. However, these results should be taken as an indication of the effectiveness of the intervention, which, given the small sample, cannot be confirmed.

Given that the COVID-19 pandemic is a situation with a high emotional impact, it seems that interventions aimed at professionals might be carried out for longer periods and that institutions should provide psychological first aid to their workers in critical situations that require it. On the other hand, although we consider that online intervention proposals may be promising, more research is needed in this regard. Future research should measure, if possible, whether face-to-face interventions improve the mental health of workers in these types of situations at the same or higher levels than the previous ones. Finally, the interpretation of our results and the recommended intervention guidelines can serve as a reference for continued testing and improving future programs of this type.

## Figures and Tables

**Table 1:** Sociodemographic characteristics of the sample.

Variables	IG (N = 17)	CG (N = 15)	*t*/χ^2^	*p*
	Mean (SD) or N (%)	Mean (SD) or N (%)		
Age	41.05 (11.07)	39.06 (12.54)	0.477	0.637
Gender			6.099	0.014 *
Man	3 (17.6%)	9 (60%)		
Woman	14 (82.4%)	6 (40%)		
Marital Status			3.032	0.220
Married	9 (52.9%)	4 (26.7%)		
Unmarried	8 (47.1%)	10 (66.6%)		
Divorced	0 (0%)	1 (6.7%)		
Education Level			1.700	0.427
Primary	0 (0%)	1 (6.7%)		
Secondary	7 (41.2%)	4 (26.7%)		
University	10 (58.8%)	10 (66.6%)		
Employment situation			4.493	0.343
Psychologist	3 (17.6%)	1 (6.7%)		
Carer	8 (47.1%)	12 (79.9%)		
Coordinator	4 (23.5%)	1 (6.7%)		
Driver	1 (5.9%)	0 (0%)		
Human Resources	1 (5.9%)	1 (6.7%)		
Workplace			3.124	0.077
Day center	3 (17.6%)	7 (46.7%)		
Residency	14 (82.4%)	8 (53.3%)		
Years of experience working with people with ID	14.29 (11.55)	10.00 (9.46)	1.140	0.263

Note: IG = Intervention Group, CG = Control Group, * *p* < 0.05.

**Table 2:** Theoretical content and objectives in the “Professionals in emotional crisis: COVID-19” program.

Session	Objective	Contents and Activities
First	Analyze the critical situation (COVID-19)	-Assessment of the COVID-19 situation in their workplace (Work/isolation, positive situation, action protocol, health of users and professionals). -Practice of full attention centered on difficult emotions meditation. -Practice ventilation/emotional relief. -Practice of how to deal with difficult emotions (“The seven steps of emotional balance”).
Second	Acceptance of the lived situation and awareness of needs	-Practice of full attention centered on compassion meditation. -Practice of the exercise “learning to know my reality”. (analysis of the emotions and thoughts they feel frequently, and which ones they avoid.) -Psychoeducation on burnout. -Awareness of needs (what they need and what can be done to get it). -Practice of full attention centered on forgiveness meditation.
Third	Provide self-care strategies to better cope with the situation experienced (part I)	-Physical and emotional self-care: general guidelines. -Changing (negative) thoughts and perspectives with cognitive restructuring. -Practice of mindfulness of breathing. -Application and practice of mindfulness in daily life.
Fourth	Provide self-care strategies to better cope with the situation experienced (part II)	-Work on self-esteem: exercises for its evaluation (“Role of your best friend and worst enemy”) and techniques to improve it (reinforce achievements, propose realistic goals, forgive mistakes, correct thinking errors, etc.). -List of pleasant activities (choice and organization, taking into account the obstacles that make it difficult). -Exercises to encourage gratitude and optimism (examples: gratitude journal, map of strengths and virtues).

**Table 3:** Results for IG and CG pre- and post-interventions.

Variable	Group	Pre Mean (SD)	Post Mean (SD)	Effect Size	Factor	*F*	*p*	Benjamini–Hochberg Adjusted *p*-Value
PTGI-SF	Control (15) Intervention (17)	29.20 (7.20) 20.13 (13.37)	27.86 (7.57) 27.20 (11.65)	0.091 0.176	Time Time x GroupGroup	2.794 5.998 2.116	0.106 0.021 * 0.070	0.420
Subescale A (GHQ-28)	Control (15) Intervention (17)	5.66 (3.26) 8.50 (3.88)	6.00 (3.68) 6.87 (4.54)	0.046 0.101	Time Time x GroupGroup	1.413 3.248 2.090	0.244 0.082 0.159	0.820
Subescale B (GHQ-28)	Control (15) Intervention (17)	6.21 (4.06) 8.33 (3.95)	6.85 (3.13) 7.46 (3.95)	0.001 0.035	Time Time x GroupGroup	0.022 0.979 1.071	0.884 0.331 0.310	1
Subescale C (GHQ-28)	Control (15) Intervention (17)	7.66 (1.23) 6.60 (2.22)	7.33 (2.12) 6.60 (2.22)	0.003 0.041	Time Time x GroupGroup	0.098 1.196 4.037	0.757 0.283 0.054	1
Subescale D (GHQ-28)	Control (15) Intervention (17)	0.93 (1.16) 0.81 (0.91)	0.80 (0.94) 0.81 (0.98)	0.003 0.003	Time Time x GroupGroup	0.091 0.091 0.036	0.765 0.765 0.850	0.850
MBI-AE	Control (15) Intervention (17)	14.86 (7.61) 18.06 (7.85)	15.20 (7.58) 19.37 (10.02)	0.033 0.012	Time Time x GroupGroup	0.983 0.348 1.632	0.330 0.560 0.212	1
MBI-DP	Control (15) Intervention (17)	4.20 (3.36) 5.93 (5.18)	4.53 (3.79) 4.87 (4.73)	0.006 0.023	Time Time x GroupGroup	0.187 0.685 0.622	0.669 0.415 0.437	0.830
MBI-RP	Control (15) Intervention (17)	39.86 (5.50) 40.06 (4.37)	40.46 (5.26) 37.93 (5.50)	0.034 0.100	Time Time x GroupGroup	1.009 3.223 0.472	0.323 0.083 0.497	0.553
CSI-PS	Control (15) Intervention (17)	16.07 (3.06) 13.75 (6.49)	15.23 (2.65) 13.62 (5.17)	0.012 0.007	Time Time x GroupGroup	0.330 0.182 1.568	0.570 0.673 0.221	0.841
CSI-SC	Control (15) Intervention (17)	6.00 (4.78) 5.28 (6.04)	9.57 (4.65) 7.42 (5.30)	0.195 0.057	Time Time x GroupGroup	6.307 1.577 0.196	0.019 * 0.220 0.662	1
CSI-EE	Control (15) Intervention (17)	9.92 (4.59) 8.73 (5.67)	11.14 (3.99) 11.66 (5.30)	0.164 0.032	Time Time x GroupGroup	5.278 0.907 0.044	0.030 * 0.3440.836	0.982
CSI-WT	Control (15) Intervention (17)	13.50 (5.44) 13.93 (3.82)	13.71 (5.46) 15.06 (4.19)	0.020 0.009	Time Time x GroupGroup	0.543 0.252 0.347	0.468 0.619 0.561	0.825
CSI-SS	Control (15) Intervention (17)	14.30 (5.07) 11.60 (5.27)	14.00 (4.32) 12.33 (5.75)	0.002 0.012	Time Time x GroupGroup	0.043 0.317 1.508	0.837 0.578 0.231	0.825
CSI-CR	Control (15) Intervention (17)	11.46 (5.01) 11.37 (3.89)	11.15 (5.42) 12.18 (4.46)	0.004 0.018	Time Time x GroupGroup	0.099 0.487 0.093	0.756 0.491 0.762	0.892
CSI-PA	Control (15) Intervention (17)	5.92 (3.14) 8.40 (4.96)	6.07 (3.60) 7.53 (5.30)	0.009 0.017	Time Time x GroupGroup	0.243 0.473 1.830	0.626 0.498 0.187	0.830
CSI-SW	Control (15) Intervention (17)	7.66 (3.59) 7.37 (4.06)	7.13 (3.70) 7.50 (4.24)	0.002 0.005	Time Time x GroupGroup	0.059 0.153 0.001	0.810 0.698 0.974	0.821
CD-RISC-10	Control (15) Intervention (17)	29.07 (3.89) 28.93 (5.99)	29.92 (3.75) 28.43 (6.23)	0.002 0.032	Time Time x GroupGroup	0.063 0.913 0.214	0.803 0.347 0.647	0.867
SLWS	Control (15) Intervention (17)	25.60 (3.88) 26.94 (3.59)	24.66 (4.70) 26.88 (3.93)	0.032 0.025	Time Time x GroupGroup	0.989 0.769 1.765	0.328 0.388 0.194	0.862
LOTR-R	Control (15) Intervention (17)	16.13 (2.92) 0.25 (3.33)	16.13 (2.74) 16.06 (3.80)	0.002 0.002	Time Time x GroupGroup	0.071 0.0710	0.791 0.791 0.984	0.832
TECA	Control (15) Intervention (17)	129.57 (7.20) 130.85 (9.64)	126.42 (8.83) 128.50 (7.05)	0.118 0.003	Time Time x GroupGroup	3.491 0.071 0.372	0.073 0.792 0.547	0.792

Notes: MBI-EE = Emotional Exhaustion (Burnout); MBI-DP = Depersonalization (Burnout); MBI-PA = Personal Accomplishment (Burnout); TECA = Cognitive and Affective Empathy Test; CD-RISC-10 = Connor–Davidson Resilience Scale; PTGI-SF = Post-Traumatic Growth Inventory Short Form; SLWS = Satisfaction With Life Scale; LOT-R = Life Orientation Test Revised (Optimism); CSI-PS = Problem Solving (Coping Strategies Inventory); CSI-SC = Self-Criticism (Coping Strategies Inventory); CSI-EE = Express Emotion (Coping Strategies Inventory); CSI-WT = Wishful Thinking (Coping Strategies Inventory); CSI-SS = Social Support (Coping Strategies Inventory); CSI-CR = Cognitive Restructuring (Coping Strategies Inventory); CSI-PA = Problem Avoidance (Coping Strategies Inventory); CSI-SW = Social Withdrawal (Coping Strategies Inventory);; Subscale A (GHQ-28) = Somatic symptoms (Mental Health); Subscale B (GHQ-28) = Symptoms of anxiety/distress and insomnia (Mental Health); Subscale C (GHQ-28) = Symptoms of social dysfunction (Mental Health); Subscale D (GQH-28) = Symptoms of depression (Mental Health); Pre = pre-evaluation; Post = post-evaluation; * *p* < 0.05.

## Data Availability

Not applicable.
